# Early Positive Fluid Balance as a Clinical Marker of Systemic Capillary Leak and Clazosentan Intolerance After Aneurysmal Subarachnoid Hemorrhage

**DOI:** 10.1007/s12028-026-02476-5

**Published:** 2026-03-24

**Authors:** Shinsuke Muraoka, Takashi Izumi, Kazuki Nishida, Basile Chrétien, Issei Takeuchi, Masahiro Nishihori, Shunsaku Goto, Ryuta Saito

**Affiliations:** 1 Department of Neurosurgery, Nagoya University Graduate School of Medicine, Nagoya, Aichi Japan; 2 Department of Biostatistics, Kyoto University School of Public Health, Kyoto, Japan; 3 Department of International Medical Education, Nagoya University Graduate School of Medicine, Nagoya, Japan

**Keywords:** Aneurysmal subarachnoid hemorrhage, Capillary leak, Clazosentan, Fluid balance, Delayed cerebral ischemia, Neurocritical care

## Abstract

**Background:**

Clazosentan, a selective endothelin A receptor antagonist, mitigates cerebral vasospasm following aneurysmal subarachnoid hemorrhage (aSAH). Although it reduces vasospasm-related events, it is also associated with adverse events (AEs) related to fluid retention, which are hypothesized to stem from systemic capillary leaks. These AEs often necessitate early discontinuation of therapy. This study aimed to identify early clinical markers of clazosentan intolerance, specifically focusing on fluid balance.

**Methods:**

This sub-analysis utilized data from the RECOVER registry, focusing on 208 patients diagnosed with aSAH who received clazosentan. Postoperative fluid status was quantified by calculating the mean daily fluid balance during postoperative days (POD) 1–3. The primary endpoint was discontinuation of clazosentan because of AEs. To identify independent predictors, a multivariable logistic regression analysis was conducted. A favorable outcome was defined as a modified Rankin Scale score of 0–2 at discharge.

**Results:**

A total of 31 (14.9%) patients discontinued clazosentan on POD 6 [interquartile range (IQR), 3–10]. Discontinuation was most frequently attributed to worsening respiratory status (13/31, 41.9%), consistent with fluid overload. During POD 1–3, fluid intake was comparable between groups, whereas urine output was lower in those who discontinued treatment, yielding an early positive balance. Spline analysis showed a sharp risk rise beyond + 750 mL/day; dichotomization at this threshold confirmed an independent association with discontinuation [≥ +750 mL/day: adjusted odds ratio (OR), 3.41; 95% confidence interval (CI), 1.29–8.92; *p* = 0.012). Discontinuation was associated with lower odds of a favorable outcome (46.7% vs. 68.6%; adjusted OR, 0.40; 95% CI 0.18–0.88; *p* = 0.023).

**Conclusions:**

Early positive fluid balance was a strong independent predictor of clazosentan discontinuation and was associated with poor functional outcomes. Early postoperative fluid balance may serve as a practical bedside marker of clazosentan intolerance and fluid-retentive physiological response, potentially involving increased vascular permeability. Goal-directed fluid management targeting euvolemia during the first 72 h may help optimize tolerability and enhance the therapeutic efficacy of clazosentan.

## Introduction

Aneurysmal subarachnoid hemorrhage (aSAH) is a severe form of stroke, in which delayed cerebral ischemia (DCI) is the primary determinant of poor long-term outcomes [1]. The pathophysiology of DCI is multifactorial, with cerebral vasospasm, driven in part by the potent vasoconstrictor endothelin-1, playing a significant role [2, 3]. Clazosentan, a selective endothelin A (ET_A_) receptor antagonist, was developed as a targeted intervention to counteract this mechanism.

The landmark Clazosentan to Overcome Neurological Ischemia and Infarction Occurring after Subarachnoid Hemorrhage (CONSCIOUS)-1 trial demonstrated that clazosentan induces a robust, dose-dependent reduction in angiographic vasospasm [4]. However, this mechanistic efficacy has not consistently translated into improved functional outcomes, highlighting a clinical paradox. In subsequent phase III trials, CONSCIOUS-2 (in surgically clipped patients) and CONSCIOUS-3 (in coiled patients), clazosentan failed to demonstrate a significant improvement in functional outcomes (Glasgow Outcome Scale Extended) despite reducing vasospasm-related morbidity and mortality in the high-dose arm of CONSCIOUS-3 [5, 6]. By contrast, pivotal phase III trials conducted in Japan showed that clazosentan significantly reduced vasospasm-related morbidity and mortality, leading to its regulatory approval in Japan and suggesting potential differences in the population or clinical practice patterns that may influence the risk–benefit profile [7]. This vasospasm–outcome dissociation remains a persistent challenge in the clinical application of clazosentan.

A plausible explanation for this translational gap lies in clazosentan’s adverse event profile. Across all major trials, clazosentan has consistently been associated with an increased incidence of pulmonary edema, pleural effusion, hypotension, and brain edema [4–6] These complications, particularly those related to fluid retention, are not minor; they often become dose-limiting and necessitate premature discontinuation of therapy, thereby negating the potential benefits of sustained vasospasm prevention [8]. A “two-hit” hypothesis, grounded in clazosentan’s pharmacodynamics, offers a mechanistic framework. The first is an aSAH-induced systemic inflammatory response that compromises the endothelial glycocalyx [9]. The second hit is clazosentan itself. Selective ET_A_ blockade leads to the unopposed stimulation of endothelial endothelin type B (ET_B_) receptors, which are hypothesized to increase vascular permeability and promote systemic capillary leakage [9]. In this vulnerable state, even standard fluid management may precipitate fluid retention and pulmonary complications, raising a critical clinical question: can early predictors of this drug intolerance be identified?

Therefore, postoperative fluid balance—an early and readily available bedside metric—may serve as a pragmatic surrogate of early clazosentan intolerance. A positive balance may reflect an exaggerated fluid-retentive response to clazosentan’s systemic effects on vascular permeability, preceding the onset of overt clinical complications. Using data from the RECOVER registry, this study aimed to test this hypothesis by (i) identifying independent predictors of clazosentan discontinuation, with a specific focus on early fluid balance as a marker of drug intolerance and (ii) evaluating the association between treatment discontinuation and functional outcomes.

## Methods

### Study Design and Clinical Setting

This retrospective multicenter cohort study was conducted as a sub-analysis of the Retrospective Study of Clazosentan and Fasudil in Subarachnoid Hemorrhage: Vasospasm Prevention and Patient Prognosis (RECOVER) registry, which collected data on patients with aSAH between April 2021 and March 2024 [10]. The present study focused on patients who received clazosentan perioperatively. No formal a priori sample size calculation was performed for this retrospective analysis; the sample size (208 patients) was determined by the available cases in the RECOVER registry. The participating centers included Nagoya University Hospital and its affiliated neurosurgical centers. The study protocol was approved by the Institutional Review Board of Nagoya University Hospital (approval no. 2022–0476), and the requirement for informed consent was waived. All study procedures were conducted in accordance with the Declaration of Helsinki, and the study reporting adhered to the Strengthening the Reporting of Observational Studies in Epidemiology guidelines.

### Patient Population

Adult patients with aSAH were eligible for inclusion if they met the RECOVER registry criteria: pre-onset modified Rankin Scale (mRS) score of 0–2, aneurysm secured within 48 h of symptom onset, and administration of clazosentan. Patients with incomplete key data were excluded. Among the 208 eligible patients, 31 discontinued clazosentan before completing the 14-day treatment protocol (discontinuation group), and 177 completed the full course (completion group).

### Treatment Protocol

All patients underwent aneurysm repair within 48 h of symptom onset. Clazosentan was initiated after aneurysm securing and administered as a continuous intravenous infusion at 10 mg/h, intended for up to 14 postoperative days unless discontinued because of adverse events. In our protocol, statins and cilostazol are used as adjunct preventive therapies against vasospasm in most patients (including low-grade SAH) unless contraindicated. Clazosentan was discontinued when treating physicians judged adverse events to be severe or progressive, including respiratory deterioration attributable to pulmonary edema and/or pleural effusion on clinical assessment and chest imaging, symptomatic brain edema, or refractory hypotension. After discontinuation, conventional fasudil therapy was initiated.

### Data Collection

Retrospective data on patient demographics, baseline neurological status [World Federation of Neurosurgical Societies (WFNS) grade, Fisher grade], aneurysm characteristics, treatment modality, and perioperative management parameters were collected. Baseline renal function was captured as the presence or absence of chronic kidney disease (CKD; estimated glomerular filtration rate (EGFR) < 60 mL/min/1.73 m^2^) when available; however, detailed creatinine/eGFR values were not consistently recorded for this registry sub-analysis. Clinical outcomes, including mRS score at discharge and complications were documented.

### Perioperative Fluid Management

Daily fluid intake and output were recorded through postoperative day (POD) 15. Intravenous or oral loop diuretics were administered intermittently to maintain euvolemia. Diuretic exposure (use and dose) was not systematically captured in the present dataset and therefore was not included in the statistical analyses. Vasopressor support was initiated in patients with hypotension and adequate urine output. In most patients during the acute intensive care unit (ICU) phase, urine output was measured using an indwelling urinary catheter (Foley) as part of routine hourly recording. After catheter removal, urine volume was measured using a dedicated urine-measurement container/cup. All intake (intravenous fluids, medications, and oral intake) and measured outputs (urine, cerebrospinal fluid drainage when present) were recorded by nursing staff per standard protocol.

### Outcome Measures

The primary outcome measure was incidence of clazosentan discontinuation. The secondary outcome was functional status at discharge, with an mRS score of 0–2 defined as a favorable outcome.

### Imaging Protocol and Evaluation

Routine magnetic resonance imaging (MRI) and magnetic resonance angiography were performed on POD 2 and 10, and additional imaging was performed as indicated. Angiographic vasospasm was defined as a ≥ 25% reduction in arterial diameter on follow-up computed tomography angiography or digital subtraction angiography. Vasospasm-related DCI was defined as a new cerebral infarct identified on MRI between POD 4 and 20, accompanied by clinical deterioration that was not attributable to other causes.

### Statistical Analysis

Data were analyzed using Python (version 3.11.4) and R (version 4.3.3). Patient characteristics were compared between the clazosentan completion and discontinuation groups using the *t*-test or Wilcoxon rank-sum test for continuous variables, and the chi-squared or Fisher’s exact test for categorical variables. Given the minimal rate of missing data (< 2% for all covariates), complete case analysis was performed without multiple imputations. Multicollinearity was assessed using variance inflation factors, all of which were < 1.5, indicating a stable estimate. Daily fluid balance was visualized as a preliminary step to compare temporal trends in fluid balance between groups during the early postoperative period. To investigate the potential nonlinear association between the mean daily fluid balance during POD 1 to 3 and clazosentan discontinuation, a natural cubic spline analysis was conducted. Subsequently, a multivariate logistic regression model was constructed to quantitatively evaluate the impact of fluid balance. The model evaluated the association between the mean daily fluid balance from POD 1 to 3 and clazosentan discontinuation, adjusting for clinically relevant variables, including age, body mass index (BMI), and WFNS grade. To further delineate the clinically relevant threshold identified by spline analysis, the mean daily fluid balance (POD 1–3) was dichotomized at the inflection point at which the risk of discontinuation began to increase exponentially. Statistical significance was defined as a two-sided *p*-value of < 0.05. Model calibration and discrimination were assessed using the Hosmer–Lemeshow goodness-of-fit test and the area under the receiver operating characteristic curve (AUC), respectively. Prespecified sensitivity analyses included (i) indexing fluid balance to body weight (mL/kg/day), (ii) adjustment for an estimated insensible water loss, and (iii) exploratory models incorporating sex and aneurysm treatment modality (clipping vs. endovascular treatment) when available.

## Results

### Patient Characteristics and Predictors of Clazosentan Discontinuation

Of the 208 patients treated with clazosentan, 31 (14.9%) discontinued the drug prematurely. The median duration of discontinuation was 6 days [interquartile range (IQR), 3–10]. At baseline, patients in the discontinuation group were significantly older (66.3 ± 14.2 vs. 61.5 ± 12.5 years, *p* = 0.046) and had a lower BMI (21.0 ± 3.5 vs. 22.8 ± 3.9 kg/m^2^, *p* = 0.014). Other baseline characteristics were comparable between the two groups (Table 1).

**Table 1:** Baseline characteristics stratified by clazosentan discontinuation status

	Discontinuation group (*n* = 31)	Completion group (*n* = 177)	*p*-value
Age (years), mean ± SD	66.3 ± 14.2	61.5 ± 12.5	0.046
Female, *n* (%)	26 (83.9)	119 (67.2)	0.081
BMI (kg/m^2^), mean ± SD	21.0 ± 3.5	22.8 ± 3.9	0.014
Smoking history, *n* (%)	7 (22.6)	52 (29.4)	0.463
Hypertension, *n* (%)	17 (54.8)	75 (42.4)	0.207
Dyslipidemia, *n* (%)	9 (29.0)	24 (13.6)	0.052
Diabetes mellitus, *n* (%)	2 (6.5)	3 (1.7)	0.228
*WFNS grade*			0.231
I–II	14 (45.2)	98 (55.4)	
III	7 (22.6)	38 (21.5)	
IV–V	10 (32.3)	41 (23.2)	
*Fisher grade*			0.454
1–2	3 (9.7)	28 (15.8)	
3	12 (38.7)	60 (33.9)	
4	16 (51.6)	89 (50.3)	

*BMI* body mass index, *mRS* modified Rankin Scale, *SD* standard deviation, *WFNS* World Federation of Neurosurgical Societies

*P*-values are from *t*-tests, Wilcoxon rank-sum tests, or chi-squared/Fisher’s exact tests

**Fig. 1:**
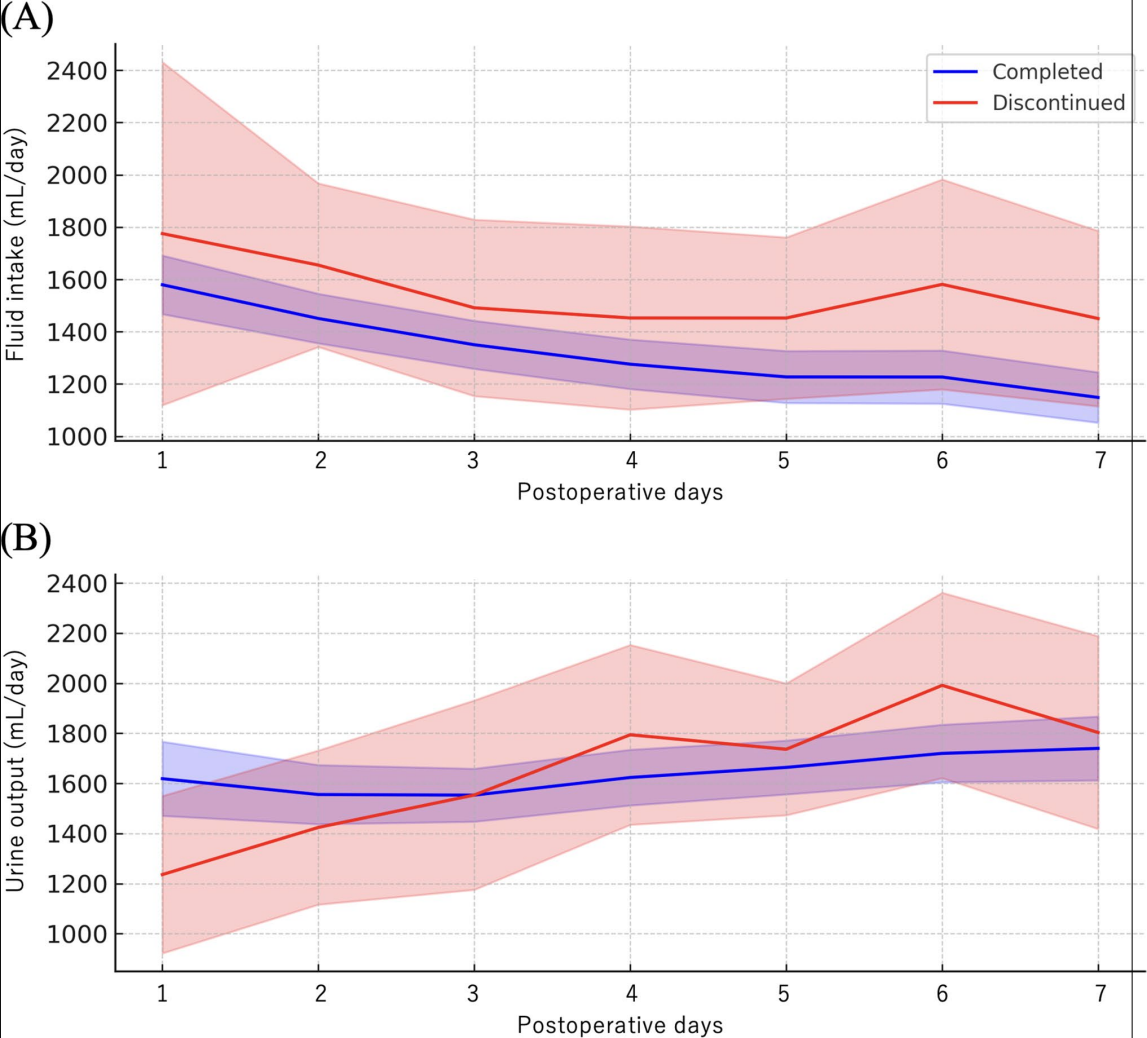
*Early postoperative fluid dynamics in completed vs. discontinued clazosentan treatment*. Daily mean fluid intake (**A**), output (**B**), and intake/output balance (intake minus output, **C**) during the first postoperative week [postoperative days (POD) 1–7] in patients who completed (blue) or discontinued (red) clazosentan therapy. The shaded areas indicate 95% confidence intervals Although the intake volumes were comparable between the groups, the output was significantly higher in the completed group during POD 1–3, resulting in an early controlled negative balance. In contrast, the discontinuation group exhibited reduced output and a progressively positive balance, which is consistent with early fluid retention. These early differences in excretory responses may underlie subsequent treatment tolerability

As shown in Fig. 1 and presented in Table 2, both groups exhibited similar trajectories of daily fluid intake during the early postoperative period (Fig. 1A). However, patients in the discontinuation group demonstrated a markedly reduced urine output on POD 1–3 (Fig. 1B), which contributed to fluid retention. In contrast, the completion group achieved a modest negative balance in the immediate postoperative phase, driven by greater output despite comparable intake (Fig. 1B). This early divergence in excretory response rather than in intake volume appears to underlie the pathophysiological mechanism that links fluid retention to treatment discontinuation.

**Table 2:** Early postoperative fluid dynamics (POD 1–3)

	Discontinuation group	Completion group	*p*-value
Mean fluid intake (POD 1–3), mL/day	1749 ± 1230	1526 ± 760	0.72
Mean fluid output (POD 1–3), mL/day	1453 ± 880	1516 ± 780	< 0.001
Mean I/O balance (POD 1–3), mL/day	+850 ± 550	−150 ± 450	< 0.001
I/O balance ≥ +750 mL/day, *n* (%)	15 (48.4)	20 (11.3)	< 0.001

*I/O* intake/output, *POD* postoperative day

### Early Fluid Balance as a Predictor of Discontinuation

**Fig. 2:**
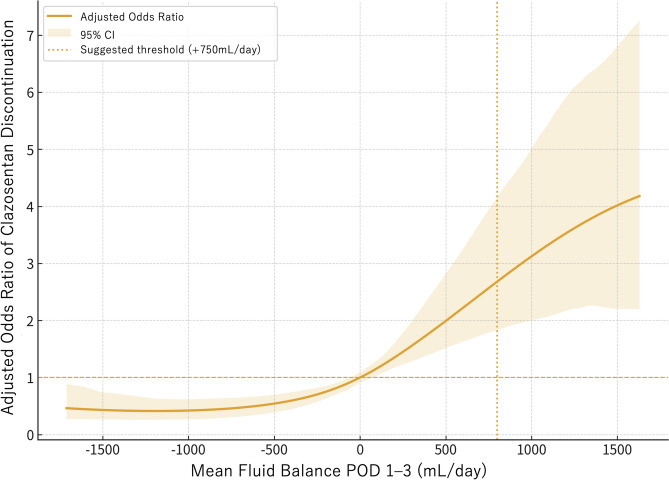
*Association between early fluid balance (POD 1–3) and clazosentan discontinuation.* A natural cubic spline model illustrates the adjusted odds ratio for clazosentan discontinuation on the basis of the mean daily fluid balance on postoperative days [(POD) 1–3]. The risk remains low with a negative or neutral balance but increases markedly beyond approximately +750 mL/day. The shaded areas represent 95% confidence intervals

Natural cubic spline analysis illustrated the relationship between the mean daily fluid balance (POD 1–3) and the probability of clazosentan discontinuation, revealing a sharp increase in risk beyond approximately +750 mL/day (Fig. 2). Patients who maintained balance below this threshold exhibited a substantially lower risk of discontinuation.

Taken together, these findings indicate that an excessive positive fluid balance may predispose patients to clazosentan discontinuation. In contrast, maintaining euvolemia or a mildly negative balance within the first 72 h after aneurysm repair represents the optimal window for drug tolerability.

In the multivariable logistic regression analysis, an early postoperative positive fluid balance ≥  +750 mL/day (mean from POD 1–3) remained an independent predictor of clazosentan discontinuation [adjusted OR, 3.41; 95% confidence interval (CI), 1.29–8.92; *p* = 0.012] (Table 3). Other covariates including age, BMI, and WFNS grade, did not reach statistical significance, and variance inflation factors < 1.5 confirmed minimal collinearity. The consistency of the effect estimates across both univariate and complete-case multivariate models supports the robustness of these findings. Given the minimal rate of missing data (< 2% for all covariates), the observed associations were unlikely to have been influenced by imputation bias or sample attrition.

**Table 3:** Multivariable predictors of clazosentan discontinuation

	Adjusted odds ratio (95% CI)	*p*-value
I/O ≥ +750 mL/day (POD 1–3)	3.41 (1.29–8.92)	0.012
Age (per 10 years)	1.25 (0.98–1.60)	0.071
BMI (per 1.0 kg/m^2^)	0.90 (0.79–1.02)	0.098

*BMI* body mass index, *CI* confidence interval, *I/O* intake/output, *POD* postoperative day

Logistic regression model using complete-case analysis

The final discontinuation model demonstrated adequate calibration (Hosmer–Lemeshow *p* = 0.45) and good discrimination (AUC = 0.80).

In sensitivity analyses, fluid balance indexed to body weight (mL/kg/day) remained a significant predictor of discontinuation, with an inflection point at approximately +12 mL/kg/day (≈ +720 mL/day for a 60-kg patient), consistent with the +750 mL/day threshold used in the primary analysis.

After subtracting a uniform estimate of insensible water loss (0.8 L/day) from measured balance, the discontinuation group remained significantly more positive than the completion group (mean +180 vs. −320 mL/day; *p* = 0.009), and the adjusted balance continued to predict discontinuation (adjusted OR, 1.45 per +250 mL/day; 95% CI, 1.10–1.92).

Baseline aneurysm treatment modality did not differ significantly between groups (clipping: 69.0% vs. 59.7%, *p* = 0.166). In an exploratory model additionally adjusting for treatment modality and sex, early positive balance remained independently associated with discontinuation (adjusted OR, 4.95; 95% CI, 2.01–12.2; *p* = 0.001), whereas treatment modality itself was not significantly associated with discontinuation.

### Treatment Discontinuation and Clinical Outcomes

Clazosentan was discontinued in several patients owing to adverse events with overlapping causes, including worsening respiratory status (13 patients), hypotension (5), brain edema (5), and other factors. Pleural effusion occurred in 15 patients (7.2%) and pulmonary edema in 13 patients (6.3%), with a median onset on POD 5 (IQR 3–8) and 4 (IQR 3–8), respectively. Brain edema was observed in 19 patients (9.1%), with a median onset on POD 3 (IQR, 2–4). Among the patients with an early positive fluid balance exceeding +750 mL/day, 66.7% discontinued treatment because of respiratory deterioration, a rate significantly higher than that observed in the neutral or negative balance groups (18.8%, *p* = 0.009) (Table 4). Patients in the discontinuation group exhibited a significantly lower rate of favorable functional outcomes at discharge (mRS 0–2) compared with the completion group (46.7% vs. 68.6%; adjusted OR, 0.40; 95% CI, 0.18–0.88; *p* = 0.023) (Table 5). These findings indicate that treatment discontinuation was associated with poorer short-term functional outcomes at discharge. When stratified by the WFNS grade, discontinuation was directly associated with a reduced likelihood of a favorable outcome across all grades, although the interaction did not reach statistical significance.

**Table 4:** Primary cause of clazosentan discontinuation based on early fluid balance

Primary cause of clazosentan discontinuation	Total *n* = 31	I/O ≥ +750 mL/day (POD 1–3) *n* = 15	I/O < +750 mL/day (POD 1–3) *n* = 16	*p*-value
Worsening respiratory status (plural effusion/pulmonary edema)	13 (41.9%)	10 (66.7%)	3 (18.8%)	0.009
Hypotension	5 (16.1%)	2 (13.3%)	3 (18.8%)	0.70
Brain edema	5 (16.1%)	1 (6.7%)	4 (25.0%)	0.18

*I/O* intake/output, *POD* postoperative day

**Table 5:** Multivariable predictors of favorable clinical outcomes

	Adjusted odds ratio (95% CI)	*p*-value
Discontinuation (vs. completion)	0.40 (0.18–0.88)	0.023
Age (per 10 years)	0.95 (0.92–0.99)	0.011
BMI (per 1.0 kg/m^2^)	1.03 (0.91–1.17)	0.640
WFNS grade 3 (vs. WFNS grade 1–2)	0.55 (0.25–1.21)	0.138
WFNS grade 4–5 (vs. WFNS grade 1–2)	0.20 (0.09–0.45)	< 0.001

*BMI* body mass index, *CI* confidence interval, *WFNS* World Federation of Neurosurgical Societies

Odds ratios and *p*-values were obtained using a logistic regression model adjusted for age, BMI, and WFNS grade

## Discussion

This study identified early postoperative positive fluid balance (≥ +750 mL/day) as an independent predictor of clazosentan discontinuation in patients with aSAH. Patients with early positive balance were more likely to discontinue because of respiratory deterioration accompanied by pulmonary edema and/or pleural effusion, and this clinical pattern is consistent with fluid-retentive intolerance to therapy. Importantly, our observational data cannot determine whether discontinuation itself contributes causally to worse outcomes; rather, discontinuation likely identifies a subset of patients who develop severe systemic complications early after aSAH. In this context, clazosentan intolerance—and the systemic physiology it reflects—may be associated with poorer short-term functional outcomes at discharge, providing one plausible framework for the long-standing “vasospasm–outcome dissociation” of clazosentan. Because clazosentan was routinely administered in this cohort as part of an aggressive vasospasm-prevention strategy, our analyses address predictors of intolerance among treated patients and may not directly generalize to settings in which clazosentan is selectively used.

### Mechanistic Interpretation of Fluid Dynamics

A three-layer analysis of intake, output, and fluid balance revealed that early fluid divergence was primarily driven by reduced output rather than excessive infusion. Despite comparable intake volumes, patients who completed clazosentan therapy demonstrated greater early diuresis and achieved a neutral-to-negative balance, whereas those who discontinued treatment exhibited a more positive balance within the first 72 h. Because urine output is a nonspecific signal that can be influenced by physiologic antidiuresis, diuretic administration, and renal function, these patterns should be interpreted cautiously; nonetheless, they are consistent with impaired early fluid clearance. One plausible interpretation is that early diuretic responsiveness may reflect preserved endothelial and renal integrity under the systemic stress of subarachnoid hemorrhage, mitigating interstitial fluid accumulation and facilitating clazosentan tolerance. Conversely, patients unable to achieve early fluid clearance may transition to a state of capillary leakage and volume overload, precipitating pulmonary edema or pleural effusion that necessitates cessation of treatment. Thus, early postoperative output dynamics, rather than the administered fluid volume, represent the pivotal determinants of clazosentan tolerability. These findings align with those of prior studies that demonstrated that early diuretic responsiveness predicts tolerance to negative fluid balance and favorable outcomes in critically ill patients [11–13]. Early postoperative diuresis may serve as a physiological marker of preserved renal and endothelial integrity, enabling safe achievement of a mild negative balance without compromising perfusion. Conversely, a blunted excretory response in the early phase, particularly under inflammatory stress, is associated with systemic capillary leakage, interstitial fluid accumulation, and pulmonary edema [14]. This interpretation is consistent with current models of endothelial permeability regulation, wherein glycocalyx degradation and tight junction disruption promote transudation of plasma fluid even under modest positive balances [15, 16]. Together, these mechanistic insights support our observation that early controlled negative balance reflects a resilient systemic state enabling completion of clazosentan therapy.

### Biological Plausibility: The “Two-Hit” Hypothesis

This observed association is grounded in the pharmacological profile of clazosentan and the systemic pathophysiology of aSAH. Clazosentan’s selective blockade of ET_A_ receptors results in the unopposed stimulation of ET_B_ receptors, a pharmacodynamic imbalance hypothesized to increase vascular permeability through multiple pathways, including enhanced nitric oxide production and modulation of tight junction proteins, promoting systemic capillary leak [9].

A “two-hit” hypothesis may explain the heightened vulnerability of patients with aSAH. The initial hemorrhage and subsequent systemic inflammatory response constitute the first hit, inducing baseline endothelial injury and degradation of the endothelial glycocalyx, a critical regulator of vascular permeability [15, 16]. This state renders the vasculature primed for injury. The second hit occurred with clazosentan administration, which further increased vascular permeability via ET_B_ receptor stimulation [17]. In this doubly compromised state, standard fluid administration in the intensive care unit may overwhelm the body’s compensatory mechanisms, leading to rapid fluid extravasation. This manifests clinically as a positive fluid balance that may progress to pulmonary edema or pleural effusion. The observation that even a modest positive balance of a few hundred milliliters per day predicts discontinuation reinforces the model of heightened susceptibility.

### Context within Modern aSAH Management Guidelines

These findings should be interpreted in the context of the evolution of fluid management strategies for neurocritical care. The era of prophylactic “Triple-H” therapy, which included hypervolemia, has been abandoned owing to a lack of demonstrated benefit and evidence of harm [18, 19]. Current guidelines from the American Heart Association/American Stroke Association and the Neurocritical Care Society now emphasize the maintenance of euvolemia and explicitly discourage hypervolemia to prevent complications such as pulmonary edema [1, 20]. This principle has been reinforced in recent reviews addressing DCI management [21].

Clazosentan therapy, if not carefully managed, may drive patients toward the very state that contemporary guidelines caution against: positive fluid balance and fluid overload. This underscores the critical clinical tension that the use of a novel agent for vasospasm prevention may inadvertently conflict with the fundamental principles of systemic critical care. The positive fluid balance observed in the discontinuation group represents not only a physiological deviation but also a departure from current standards of care and a potential marker of iatrogenic harm.

### Reinterpreting the CONSCIOUS Trials

This framework offers a new perspective on the contradictory findings of the CONSCIOUS-2 and CONSCIOUS-3 trials. Although these studies recommended a minimum fluid intake, they did not mandate a strict, protocolized, goal-directed strategy to maintain euvolemia [4–6] Notably, in the major phase III trials (CONSCIOUS-2, CONSCIOUS-3, and REACT), approximately 90% of patients in both the clazosentan and placebo arms received nimodipine as part of standard care [5, 6, 22]. Therefore, these trials did not evaluate clazosentan against a true placebo but rather assessed its additive effect within the framework of established baseline therapy with nimodipine. This context is crucial, as it may have masked a substantial treatment effect or influenced the adverse event profile.

Variations in fluid management across the participating centers may have acted as a significant, unmeasured confounder. This interpretation is supported by real-world data from multicenter cohorts, which also highlight the complexity of outcomes when clazosentan is administered alongside other therapies [23–25]. Patients treated at centers employing more liberal fluid strategies may have been driven into a positive fluid balance, precipitating adverse events that offset the therapeutic benefits. Conversely, patients managed with strict euvolemic control may experience clinical improvements. Therefore, the overall neutral effect on functional outcomes may represent an average of the opposing influences. These findings suggest that the full therapeutic potential of clazosentan is likely realized in a tightly regulated physiological environment that maintains euvolemia.

### Clazosentan Dose and Endothelin Biology

Clazosentan is generally administered as a fixed dose regardless of body weight. Although this simplifies real-world use, interindividual variability in exposure may be influenced by body size, age, and sex, and could interact with endothelin biology to shape both efficacy and intolerance signals observed in observational cohorts.

Endothelial ET_B_ receptors contribute to nitric oxide-mediated vasodilation and to clearance of circulating ET‑1. Human endothelial cell data suggest that endothelial ET_B_ receptor expression is attenuated after menopause, a change that plausibly shifts the balance toward ET_A_-mediated vasoconstrictor tone and endothelial dysfunction [27]. Aging-related vascular dysfunction has also been linked to upregulated ET‑1 signaling and reduced nitric oxide bioavailability [28, 29], and venous endothelial ET_B_ receptor protein expression appears to be preserved with aging in men [30], underscoring potentially divergent aging trajectories by sex. Taken together, these observations provide a biologically plausible (but indirect) explanation for the female predominance in the discontinuation group in our cohort and support considering sex- and age-related endothelin biology when interpreting clazosentan-associated fluid retention.

### Clinical Implications

These results have important practical implications. Routine, meticulous monitoring of daily fluid balance and body weight is not ancillary but serves as a vital early warning system during clazosentan infusion. An early and persistent positive fluid balance should be considered a red flag, warranting prompt reassessment. The completion group achieved a mean early balance of −150 ± 450 mL/day, setting a critical physiological benchmarking target for safe clazosentan continuation. Clinicians should aim to maintain a neutral or mildly negative balance during the early phase. An early and persistent positive fluid balance ≥  +750 mL/day should be considered as a red flag, warranting prompt reassessment. This may include intensive diuretic therapy or, in high-risk patients, the implementation of advanced hemodynamic monitoring such as transpulmonary thermodilution for quantitative assessment of extravascular lung water (EVLW). EVLW provides an objective, early measure of pulmonary congestion to guide aggressive fluid de-escalation via loop diuretics before overt respiratory failure necessitates clazosentan withdrawal [24–26]. If a positive balance cannot be corrected within the first 72 h, the risk–benefit profile of continuing clazosentan should be re-evaluated, particularly before the onset of irreversible pulmonary complications.

### Limitations

This study has several limitations. First, fluid balance derived from recorded intake and output did not account for dietary water content, insensible water losses, or day-to-day variation in these components. Although our sensitivity analysis incorporating an assumed insensible loss supported the robustness of the association, future studies should combine intake/output data with serial body weight measurements and, where feasible, objective hemodynamic or pulmonary congestion assessments.

Second, the observational design precludes causal inference. Unmeasured or incompletely measured confounders may have influenced both fluid balance and the decision to discontinue clazosentan, including baseline cardiac function, early postoperative respiratory status, inflammatory burden, detailed renal indices (including baseline creatinine/eGFR), and the dosing and timing of diuretics and vasoactive agents. Because oliguria and positive balance can reflect physiologic antidiuresis, SIADH/CSWS spectrum physiology, or renal dysfunction, our results should be interpreted as hypothesis generating rather than definitive evidence of a single mechanism.

Third, we did not directly measure endothelial permeability, capillary leak biomarkers, echocardiographic indices, lung ultrasound findings, or other physiological markers that could distinguish cardiac from noncardiac causes of pulmonary edema. Therefore, the proposed link between early fluid retention and systemic capillary leak remains indirect.

Finally, outcomes were assessed at discharge only, and longer-term functional status (e.g., 90 days) was not available in this registry. The sample size, particularly of the discontinuation subgroup, limited the number of covariates that could be incorporated without overfitting. Prospective studies with standardized monitoring and long-term follow-up are warranted to validate these findings and define clinically actionable thresholds.

## Conclusions

Early positive postoperative fluid balance is a strong and independent predictor of clazosentan discontinuation in patients with aSAH. Treatment discontinuation serves as a robust marker of unfavorable functional outcomes. These findings suggest that an early positive fluid balance may act as a critical warning signal, identifying patients on a high-risk trajectory and potentially reflecting intolerance to the drug’s systemic effects on vascular permeability. Because baseline creatinine/eGFR and detailed diuretic dosing were not systematically available, mechanistic interpretation remains indirect and these findings should be interpreted as hypothesis generating. Meticulous fluid management, anchored in the principle of maintaining euvolemia, as recommended by current guidelines, is paramount for the safe administration of clazosentan and for unlocking its therapeutic potential. Prospective studies are warranted to determine whether a protocolized goal-directed euvolemic strategy can reduce discontinuation rates and translate the established anti-vasospasm efficacy of clazosentan to improved functional independence in patients with aSAH.
